# Ileocecal Intussusception in the Era of Coronavirus Disease 2019 (COVID-19) Infection and Multisystem Inflammatory Syndrome in Children (MIS-C): A Case Report and Literature Review

**DOI:** 10.7759/cureus.62731

**Published:** 2024-06-19

**Authors:** Niharika Alla, Thaer Abdul-Hadi, Florentina Litra

**Affiliations:** 1 Pediatrics, University of Florida, Pensacola, USA; 2 Pediatrics, Ascension Sacred Heart Pensacola, Pensacola, USA

**Keywords:** gastrointestinal symptoms, kawasaki disease, mis-c, acute covid-19, intussusception

## Abstract

Ileocecal intussusception (ICI) is the most common abdominal emergency and cause of intestinal obstruction in young children, carrying a high risk of mortality and morbidity. Enteric viral infectious and inflammatory syndromes are known triggers for intussusception (ileoileal and ileocolic) by causing mesenteric lymphoid hyperplasia that may act as a leading point allowing the bowel to invaginate into itself. Gastrointestinal (GI) symptoms are common in children with coronavirus disease 2019 (COVID-19) infection, with a subset of patients solely having GI complaints at the time of presentation.

COVID-19 as a trigger for intussusception in children has been hypothesized and suggested in multiple cases reported to date, both during the acute phase of illness and as a part of multisystem inflammatory syndrome in children (MIS-C). We present a seven-month-old male who developed ICI and became a diagnostic dilemma due to viral co-infections and the gradual emergence of MIS-C during the hospital stay. We are describing this presentation in an attempt to expand the understanding of the implications of COVID-19 and MIS-C in this young and unique age group.

## Introduction

Ileocecal intussusception (ICI) is the most common abdominal emergency and cause of intestinal obstruction in young children, especially in infants in the first year of life [1]. Without timely identification, ICI can lead to many critical complications, including bowel necrosis, perforation, shock, and multi-organ failure, with the possibility of death. Viral infectious diseases and inflammatory conditions can trigger intussusception (ileoileal and ileocolic) by causing mesenteric lymphoid hyperplasia that may act as a leading point allowing the bowel to invaginate into itself [2]. Coronavirus disease 2019 (COVID-19) refers to the infection caused by the novel 2019 coronavirus strain, severe acute respiratory syndrome coronavirus 2 (SARS-CoV-2), that emerged in late 2019 and has been subsequently declared a global pandemic by the World Health Organization (WHO) on March 2, 2020. As of June 21, 2023, there have been 768,187,096 confirmed cases of COVID-19, including 6,945,714 deaths, and the United States accounts for 103,436,829 confirmed cases with 1,127,152 deaths [3]. Although known for targeting the respiratory tract, COVID-19 has also resulted in many non-respiratory manifestations, affecting many organs, with gastrointestinal (GI) symptoms such as diarrhea and vomiting being common findings (13.4% and 6.3%, respectively), with a subset of patients only having such complaints at the time of presentation [4,5].

COVID-19 as a trigger for intussusception in children has been hypothesized and suggested in multiple cases reported to date [6,7]. There has also been a death documented in a 10-month-old girl who had intussusception with resultant multi-organ failure [8]. We present a seven-month-old male who developed ICI in the scope of multisystem inflammatory syndrome in children (MIS-C) and was positive for COVID-19 by nasopharyngeal polymerase chain reaction (PCR) testing. We are describing this presentation in an attempt to expand the understanding of the implications of COVID-19 and MIS-C in this young and unique age group. 

## Case presentation

A seven-month-old male initially presented to the emergency department (ED) for the evaluation of three days of fever, vomiting, and decreased urine output. A respiratory pathogen panel by PCR tested positive for human rhinovirus/enterovirus and COVID-19. He was observed in the ED for a few hours, had no further vomiting episodes, was diagnosed with viral gastroenteritis, and was discharged home with instructions for symptomatic management including antipyretics, antiemetics, and oral rehydration. He returned to the ED two days later, as he continued to have emesis with every feed, now bilious despite being on ondansetron. He also had six days of fever with a maximum temperature of 101°F and developed a rash on the buttocks and diaper area. He has had no bowel movements for the last five days.

On examination, he was febrile with a temperature of 38.2°C and tachycardic (164 bpm) with a normal respiratory rate of 46/min, an O2 saturation of 100%, and a blood pressure of 107/52 mmHg. The pertinent positive findings were a sunken fontanelle, a strawberry tongue, dry lips with a red border, an erythematous dry rash on the cheeks and forehead, a distended abdomen with no bowel sounds, and a desquamating rash noted on the bilateral buttocks. He was irritable but not lethargic. 

The initial labs were significant for mild leukocytosis; elevated blood urea nitrogen (BUN) and creatinine; elevated alanine transaminase (ALT), aspartate transaminase (AST), and lactate dehydrogenase (LDH); elevated inflammatory markers (erythrocyte segmentation rate (ESR), C-reactive protein (CRP), D-dimer, procalcitonin); normal creatinine kinase (CK), ferritin, and troponin-I; and normal coagulation studies (Table 1). The rest of the comprehensive metabolic panel (CMP) including electrolytes was normal. Urinalysis was significant for ketones, but negative for signs of infection.

**Table 1:** Lab results on day 1 and day 3

Lab (units)	Normal value	Day 1	Day 3
White blood cell count (K/uL)	6-11	12.3	8
Hemoglobin (g/dL)	10.5-13.5	11.7	9.6
Platelets (K/uL)	200-400	841	616
Erythrocyte segmentation rate (mm/hr)	0-10	25	-
Prothrombin time (seconds)	11.6-15	15.1	13.1
International normalized ratio	<4	1.2	1
Partial thromboplastin time (seconds)	23-40	27.6	27.1
D-dimer (mcg/mL)	≤0.49	9.18	3.84
Fibrinogen (mg/dL)	200-400	435	37
Antithrombin 3 (%)	82-122	-	90
Creatinine (mg/dL)	0.31-0.53	0.61	0.40
Albumin (gm/dL)	3.8-5.4	3.7	2.6
Alanine transferase (IU/L)	11-33	131	202
Aspartate transferase (IU/L)	20-67	94	71
Ferritin (ng/mL)	21.8-274.7	187.9	134.1
Lactate dehydrogenase (IU/L)	125-220	631	667
Creatinine kinase (IU/L)	30-200	110	-
Brain natriuretic peptide (pg/mL)	10-100	-	345.3
Troponin (ng/mL)	0.010-0.033	0.010	0.001
C-reactive protein (mg/dL)	≤0.90	11.76	4.66
Procalcitonin (ng/mL)	0.00-0.10	0.92	0.16

The patient had a passage of bloody stool in the ED, which prompted an abdominal ultrasound (US) that showed no evidence of intussusception. Biliary sludge was present within the gallbladder, without evidence of cholelithiasis or acute cholecystitis. The child was given a normal saline (NS) bolus and admitted for further diagnosis work-up and management. 

**Figure 1:**
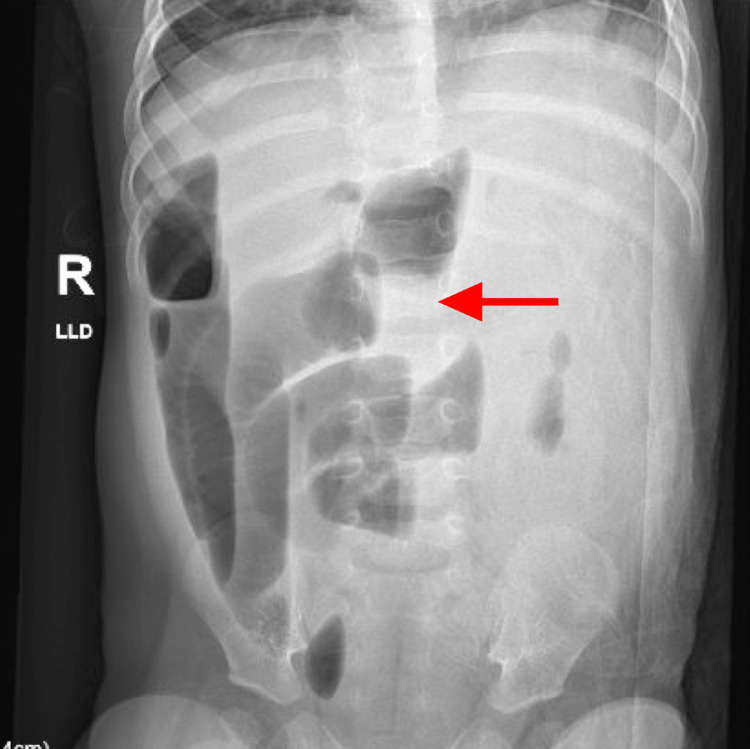
X-ray of the abdomen: AP view Numerous abnormally dilated gas and fluid distention loops of the small bowel throughout the abdomen consistent with small bowel obstruction AP: anteroposterior

**Figure 2:**
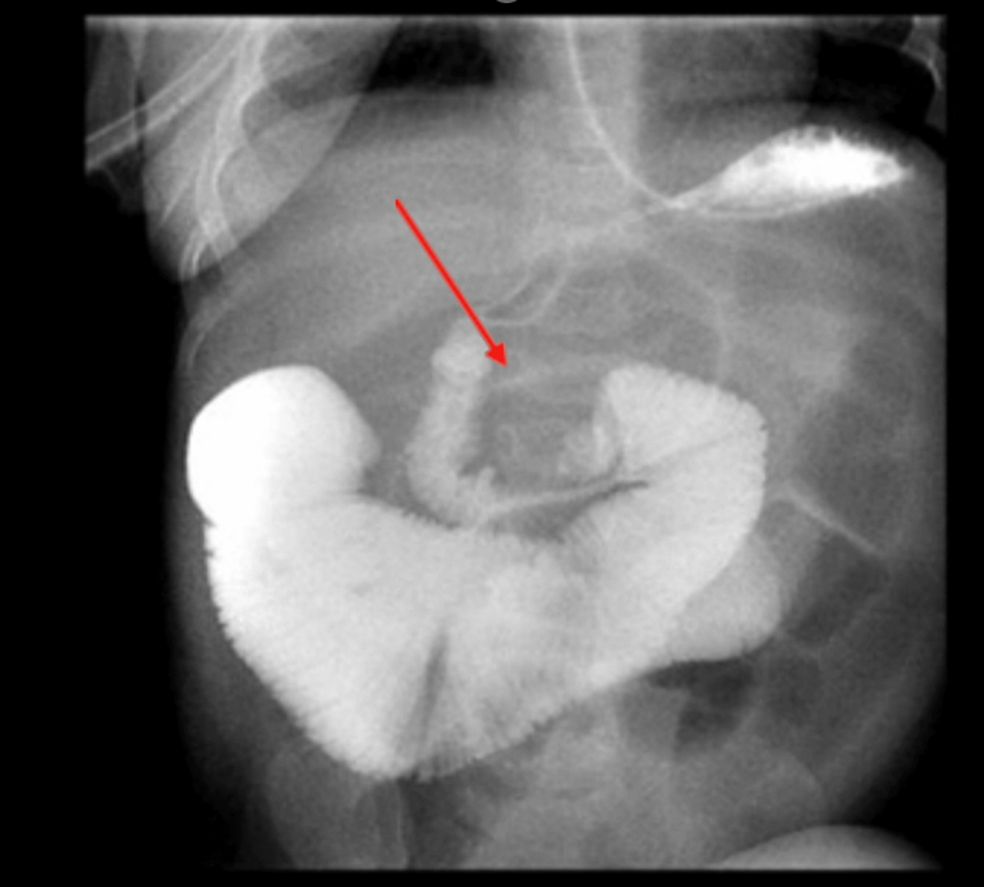
Upper GI series The fourth portion of the duodenum is somewhat inferiorly displaced. Contrast continues to flow into the proximal jejunum which is severely distended. Several distended loops of small bowel GI: gastrointestinal

**Figure 3:**
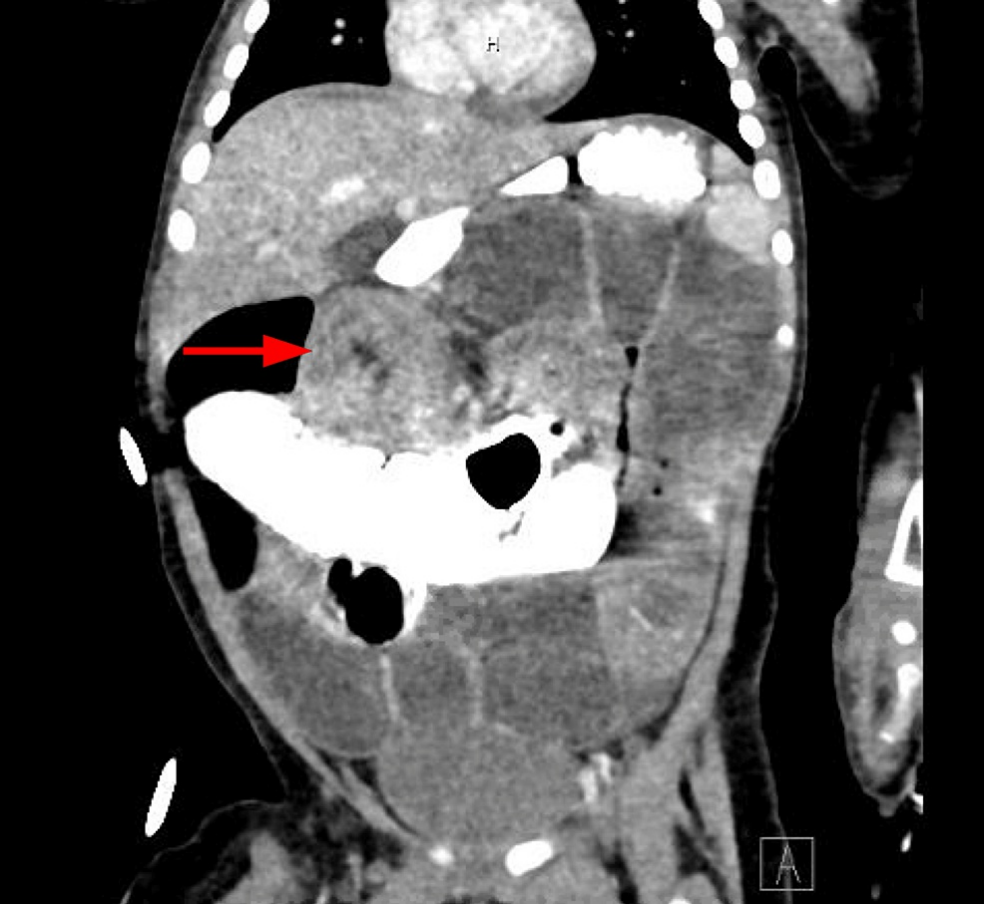
CT of the abdomen with contrast Concentric ring structure measuring 3.5 cm in size in the subhepatic region, concerning for ileocolic intussusception CT: computed tomography

After admission to the floor, the labs resulted in the following: COVID IgG positive, brain natriuretic peptide normal, and Hemoccult positive. An X-ray of the abdomen was obtained which showed small bowel obstruction (Figure 1). As the abdominal US done in the ED appeared to be normal, an upper GI series X-ray was then performed and found abnormal with several distended loops of small bowel with no definite evidence of malrotation, though the duodenum was not entirely normal in positioning, concerning for volvulus as one of the differentials (Figure 2). These findings were discussed with the pediatric surgeon, and the decision was made to proceed with a contrast computed tomography (CT) scan to further define whether this represented an atypical malrotation, volvulus, ileus, or other abnormality. CT of the abdomen showed findings of numerous abnormally dilated small bowel loops throughout the abdomen and pelvis, with many air-fluid levels; the colon appears completely decompressed and is difficult to trace throughout its length; the cecum and ascending colon are not discretely demonstrated in the right lower quadrant. The lower right quadrant contains numerous dilated small bowel loops. Small portions of the distal descending colon and sigmoid colon are shown, containing some air, in the left lower quadrant which could reflect ileocolic intussusception in the right upper quadrant of the abdomen, producing small bowel obstruction(Figure 3). Air contrast enema was recommended; an intussusception was encountered at the level of hepatic flexure which was extremely difficult to reduce. Five sessions of colonic insufflation with intermittent breaks were performed. Additionally, air was suctioned from the distended small bowel loops via the nasogastric tube. 

**Figure 4:**
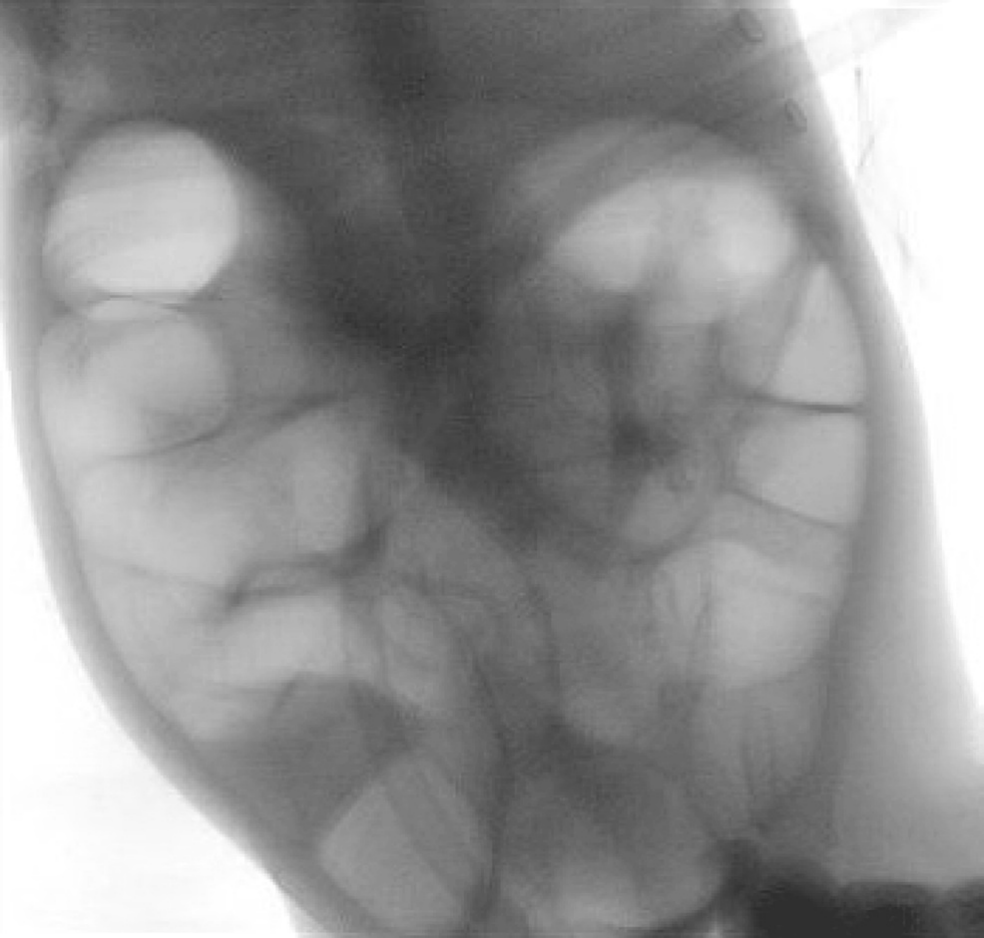
Air contrast enema Post-intussusception reduction images

After the fifth attempt at air contrast enema, intussusception was successfully reduced with help from the pediatric surgery team (Figure 4).

We diagnosed this patient with MIS-C with Kawasaki features based on the lab findings of leukocytosis, transaminitis, elevated inflammatory markers (ESR, CRP, D-dimer, procalcitonin), and the clinical signs (prolonged fever with mucosal involvement, desquamating skin rash). 

IVIG 2 g/kg was given, and methylprednisolone 1 mg/kg BID was started. An echocardiogram was done and showed normal coronary arteries and ventricular function. The IVIG was completed in the evening of hospital day 2 (HD#2). He was observed for 36 hours after completion and had stable vital signs. The methylprednisolone was switched to oral prednisolone and tapered over four weeks due to MIS-C concerns. He was also started on low-dose aspirin 5 mg/kg on HD#3 when he did not have any other active signs of bleeding. 

Pertinent lab results from HD#3 are shared in Table 1 and showed the following: complete blood count (CBC) with mild anemia and thrombocytosis, CMP normal except for ALT/AST mildly elevated, ferritin downtrending, LDH elevated, BNP elevated, inflammatory markers CRP, procalcitonin, ESR, and D-dimer all improved, troponin I normal, and coagulation studies and fibrinogen normal. Urine culture and blood culture remained negative. 

An echocardiogram was repeated on HD#3 which showed grossly normal biventricular systolic function and normal coronary arteries and an ejection fraction (EF) of 80% by M-mode and 61% by Simpsons' mode. A small pericardial effusion was also noted and reviewed with cardiology and not concerning. Due to excessive fussiness and episodic crying, an abdominal US was repeated on HD#3 and was negative for ileocolic intussusception. 

On HD#4, he was stable, afebrile, and tolerating PO well, and his rash was also resolving. Discharge planning included steroid weaning over four weeks, low-dose aspirin for four weeks, and follow-up with cardiology with a repeat echocardiogram in two weeks, which was normal. 

## Discussion

ICI is one of the most common abdominal emergencies in children younger than two years of age, with a well-known association with viral triggers including adenoviruses, enteroviruses, parechoviruses, and noroviruses [9]. 

Initially, COVID-19 was believed to be less encountered in children and not as virulent as in adults. However, reports from Europe and the United States have emerged depicting an association between COVID-19 infection in children and a delayed multisystemic inflammation that was referred to as MIS-C [10]. 

The pathogenesis of MIS-C is manifested by increased cytokine production and the overactivation of T cells and plasmablasts leading to a widespread inflammatory response that involves many systems including the GI tract [11].

COVID-19 was reported to be associated with other acute GI manifestations in the pediatric population which include mesenteric fat inflammation, intestinal wall inflammation, peritoneal effusion, abdominal lymphadenopathy, appendicitis, pancreatitis, and abdominal abscess/fluid collection [12]. Rare presentations which include pneumatosis intestinalis, pneumoperitoneum, and large-volume ascites have also been reported [13]. A few similar cases with an acute COVID-19 infection not associated with MIS-C have been reported to be complicated by intussusception [14,15].

With the emergence of COVID-19, there have been very few reported cases of intussusception as a manifestation and presentation of COVID-19, suggesting GI tract involvement by COVID-19 [13]. In this case, we have a patient presenting with isolated GI symptoms and findings of MIS-C. This raises suspicion of SARS-CoV-2 directly affecting the GI tract, leading to mesenteric adenitis and resulting in intussusception.

In a few cases where the lead point was identified, lymphoid hyperplasia of Peyer's patches within the ileum was the most common instigating lead point [16]. Although the pathogenesis of COVID-19-related intussusception is not fully understood, it can be inferred to parallel the other viral-associated intussusception cases as described as lymphoid hyperplasia of Peyer's patches and mostly within the ileum leading to an alteration in the peristaltic movement of the bowel wall which enables the telescoping of adjacent loops of the bowel into each other, manifesting as intussusception [16,1]. Notably, the widespread immune activation that has been shown to be prevalent in COVID-19 may result in hypertrophy of the intestinal Peyer's patches, thereby inciting intussusception, which could be possible in this case with the patient having MIS-C. 

Furthermore, the intestinal epithelial cells express angiotensin-converting enzyme 2 (ACE-2) receptors and the transmembrane protease serine 2 (TMPRSS2) enzyme which is hypothesized to mediate the entry of SARS-CoV-2 to intestinal cells causing the reported GI manifestations [17]. Enterocyte neuronal cells also express ACE-2 receptors, which might also explain COVID-19 GI infection [17]. The patient that we described developed intussusception in the context of MIS-C while still having positive COVID-19 and enterovirus PCR tests. In this case, it is arguable that an acute infection by COVID-19 or enterovirus could have led to the symptoms of gastroenteritis leading to intussusception; however, the timeline of symptom worsening and the laboratory findings were supportive of MIS-C [13]. Also, in retrospect, the quick resolution of GI symptoms with IVIG and steroid treatment is more suggestive of MIS-C and COVID-19 infection as the main etiology.

A study from Turku University Hospital, Finland, conducted from August 2001 to May 2022 has also shown that it can take 2-5 weeks for the enteroviruses to clear from the respiratory secretions [18]. PCR testing in general, while highly sensitive and specific, cannot distinguish between a live virus and remnant genetic material. It is possible that in our case the patient had persistent positive PCR tests as the rest of the lab work and presentation is more consistent with MIS-C [13,18]. 

Per the criteria for diagnosis of MIS-C by the American College of Rheumatology, our patient has MIS-C with the clinical manifestations of prolonged fevers (>6 days) associated with rash, oral mucosal changes, GI symptoms, and laboratory findings of CRP >3 mg/dL, COVID-19 IgG positive, and elevated BNP, procalcitonin, D-dimer, LDH, and fibrinogen [13]. 

Our theory that MIS-C might be the trigger for intussusception is also suggested by the reported association between Kawasaki disease (KD) and intussusception that can be explained by intestinal wall vasculitis with small bleeds acting as leading points for bowel telescoping [19]. MIS-C and KD share a similar presentation with the pathophysiology for both including a widespread inflammation, which supports our theory that this case of intussusception might be in fact a result of MIS-C [13]. 

In most cases, the patients meeting the diagnostic criteria of MIS-C also meet the diagnostic criteria for KD. In our case, the patient did have KD features which include mucocutaneous involvement and age of presentation being before five years [20]. 

The standard of treatment for MIS-C is immunomodulatory therapy which includes IVIG and steroids. For refractory cases, anakinra is used, and it can also be used in patients with contraindications to long courses of glucocorticoids. Infliximab may also be used for intensification therapy or as a steroid-sparing agent in children with MIS-C without evidence of macrophage activation syndrome (MAS). Serial monitoring of laboratory tests and cardiac assessment should be done as in our case [13]. 

Given the KD features, aspirin 80 mg/kg was also started in our patient after there were no signs of active bleeding [13]. 

As far as the management of uncomplicated intussusception, the gold standard is reduction by air enema, which was done in our case. In recurrent or refractory cases, surgical treatment is the next step [17].

## Conclusions

We described a case of intussusception in a patient who met the criteria for MIS-C diagnosis. This case is interesting because of the underlying diagnostic challenge. MIS-C can lead to many GI manifestations including vomiting, diarrhea, and severe cramping pain. The patient was also positive for enterovirus and COVID-19 by PCR, making it difficult to attribute the pathogenesis of intussusception to MIS-C solely, considering the concurrent viral infection and the known association between viral infections and intussusception. 

The diagnosis of intussusception was initially considered but possibly missed in this patient considering that the initial abdominal US was negative. It may have been an intermittent phenomenon initially and progressed to persistent ICI which was eventually captured on CT of the abdomen. Thankfully, the intussusception was reduced without any complications or bowel compromise. COVID-19 was linked to many described cases of intussusception, but this case is unique considering that intussusception occurred during the course of MIS-C. 

Intussusception should be highly suspected and ruled out in any conditions associated with inflammation of the gut (infectious, post-infectious, etc.). Intussusception can spontaneously resolve and may not be captured on US; therefore, recurrence or worsening of symptoms should further raise the suspicion of small bowel obstruction. Abdominal US remains the least invasive and time-consuming method of diagnosis for intussusception and should be considered before upper GI series and CT of the abdomen. A timely diagnosis can have positive repercussions on gut viability and may also lead to avoiding unnecessary invasive imaging and diagnostic procedures such as exploratory laparotomy. Additional diagnoses (KD, MIS-C, etc.) should be considered if the inflammatory state is persistent, and adequate therapies should be employed. 
